# Supplementary material for: Photosynthetic oxygen bubble stream sounds from aquatic macrophytes, and their consequences for acoustic biodiversity inventories and acoustic communication in shallow freshwater settings
Source: PLoS One. 2025 Apr 23;20(4):e0317424. doi: 10.1371/journal.pone.0317424 (PMC12017830; doi:10.1371/journal.pone.0317424)
Supplement: Attachment — Submitted filename: PLOS One review - Oct.docx [file pone.0317424.s001.docx]

**Summary**

This is an interesting piece of research that quite rightly highlights the importance of sound production by submerged aquatic plants and provides a description of sound production by *Stuckenia pectinata*. However, I feel that there are some key pieces of information missing from the manuscript that should be added before publication. For example, I think that the introduction could be expanded slightly to include a few key papers (see suggestions below). Also, more detail is required in the methods section in general, and some methods appear in the results section (e.g., aquarium recordings and the use of gas chromatography) but are not described in the methods section. You mention that the recordings were uploaded to Xeno Canto in the results - I would also put this in a Data Availability Statement at the end of the manuscript. I also think that the discussion and conclusion could be improved by thinking about the wider implications for monitoring aquatic plant sounds. These could include the use of aquatic plant sounds as indicators of pollution events / measuring the primary productivity of an ecosystem. Also, many macrophytes are indicator species and are used to assess the ecological condition of an ecosystem. Perhaps the ability to identify key macrophyte species’ sounds will allow for the assessment of freshwater ecosystems using passive acoustic monitoring?

**Abstract**

4: Most *of the* work on freshwater soundscapes.

11: What are these important consequences for acoustic bioinventories exactly?

**Introduction**

19 - Freshwater is one word (check for other instances throughout).

25 - Following “with some exceptions… add the following references that include descriptions of plant sounds in freshwater habitats:

Kratochvil, H. G., & Pollirer, M. (2017). Acoustic effects during photosynthesis of aquatic plants enable new research opportunities. *Scientific Reports*, 7(1), 44526.

Greenhalgh, J. A., Genner, M. J., & Jones, G. (2023). Diel variation in insect‐dominated temperate pond soundscapes and guidelines for survey design. *Freshwater Biology*, *68*(7), 1148-1160.

31: This is such a cool fact! I had no idea… Double check this reference though - It doesn’t seem to include anything on beetles. Perhaps this is better? <https://www.tandfonline.com/doi/abs/10.1080/01650424.2011.572558>

48: avoid ‘numerous’ and state the total number of hours/sites etc.

48: The hypothesis / research aim should be more clearly defined than “To get a better sense of the soundscapes in shallow fresh water”. Is the aim of the manuscript just to describe pondweed sounds, or to describe the entire soundscapes of these lakes? It would be good to include some analysis of the water boatman sounds if you are taking the soundscape approach.

**Results**

65 - 67: This sentence should be moved to the discussion.

69: This should be moved to the discussion, also add a recent view here that suggests >7,000 aquatic insect species are likely to produce sound globally: Desjonquères, C., Linke, S., Greenhalgh, J., Rybak, F., & Sueur, J. (2024). The potential of acoustic monitoring of aquatic insects for freshwater assessment. Philosophical Transactions of the Royal Society B, 379(1904), 20230109.

79: Can you explain how the aquarium recordings were made? How big was the tank? Where did you put the hydrophone? How did you ensure that no animals from the lake that might also produce sounds were still attached to the plant?

81: What were the other gases other than oxygen detected by the gas chromatography?

150: Please consider uploading your sounds to The Freshwater Sounds Archive and be included as a co-author in an upcoming data paper: <https://fishsounds.net/freshwater.js>

**Methods**

Need to include methods regarding the laboratory recordings, gas chromatography, and making the acoustic measurements (peak frequencies etc).

207: Add sampling rate and gain used.

207: How long did you record for? Can you be specific about the times of day that the recordings were made at? (Perhaps by checking the .wav file name time stamp?)

213: Spectrograms, not Spectrographs (check for other instances throughout). Also add FFT size and % overlap used to generate the spectrograms.

**Discussion**

155: Avoid “at our study site” and put something like: The shallow water soundscape of Lake Gull in Alberta was dominated by….

156: Personally, I am not a fan of making things into acronyms unless it’s absolutely necessary. I recommend removing them in favour of clear and simple language, but this is a stylistic thing and your choice.

159: Maybe add some clarity here and state that you think the bubbles are produced by incisions made in the leaf structure by aquatic insects, such as plant breathers (if this is indeed what you mean). Did you see this happening? It would be great to prove this. Or perhaps you mean by damage sustained by the plant? If this is the case then is it best to describe the bubbles as photosynthetic bubbles?

171: keep the referencing style the same.

173: The use of acronyms is confusing here. Make a clear distinction when you are talking about sounds produced by fish and sounds produced by plants.

187: See The Freshwater Sounds Archive: <https://fishsounds.net/freshwater.js>

190: Add Greenhalgh, J. A., Genner, M. J., & Jones, G. (2023). Diel variation in insect‐dominated temperate pond soundscapes and guidelines for survey design. *Freshwater Biology*, *68*(7), 1148-1160.

200: How about the potential to use aquatic plant sounds as indicators of pollution events / measuring the primary productivity of an ecosystem. Also, many macrophytes are indicator species and are used to assess the ecological condition of an ecosystem. Perhaps the ability to identify key macrophyte species’ sounds will allow for the assessment of freshwater ecosystems using passive acoustic monitoring?

216: Can you be more specific about how these variables were calculated? Were they calculated in Raven 2? What tool did you use, to what decimal place etc…

**Tables**

Include the species name in table legend. Add standard error values for each measurement.

**Figures**

I think that the frequency range on the y axis should be standardised across all spectrograms to allow for direct comparison. Also, each figure legend needs to include the FFT size and overlap parameters used to create them. No need to explain the axis of each figure, the units do that for us.

Do you have any pictures of the pondweed and the lakes you sampled? It would be great to see what they look like.
