# Supplementary material for: Photosynthetic oxygen bubble stream sounds from aquatic macrophytes, and their consequences for acoustic biodiversity inventories and acoustic communication in shallow freshwater settings
Source: PLoS One. 2025 Apr 23;20(4):e0317424. doi: 10.1371/journal.pone.0317424 (PMC12017830; doi:10.1371/journal.pone.0317424)
Supplement: Attachment — Submitted filename: Response to Reviewers.docx [file pone.0317424.s002.docx]

**ONE-D-24-33566**

**Response to Reviewers**

Dear Dr. Martin

Thank you for the opportunity to submit a revised draft of the manuscript “Photosynthetic oxygen bubble stream sounds from aquatic macrophytes, and their consequences for acoustic biodiversity inventories and acoustic communication in shallow freshwater” for publication in PLOS ONE. We appreciate the time and effort that you and the reviewers dedicated to providing valuable feedback on our manuscript. We have incorporated most of these suggestions into the revised manuscript. These changes are visible in the tracked changes version of the manuscript.

Please see below, in blue, a point-by-point response to the comments. Line numbers are included for the location of these changes based on the manuscript file.

Sincerely,

Katie Campbell

kgc2@ualberta.ca

University of Alberta

**Response to Editor**

1. Please ensure that your manuscript meets PLOS ONE's style requirements, including those for file naming.

**Author response:** All formatting of the paper has been completed as per the journal’s requirements.

2. In your Methods section, please provide additional information regarding the permits you obtained for the work. Please ensure you have included the full name of the authority that approved the field site access and, if no permits were required, a brief statement explaining why.

**Author response:** For the data collected, no permits were required for the recording of soundscapes. This is a non-invasive data collection technique which did not require any permits at the Summer Village of Gull Lake, Alberta, Canada.

3. Thank you for stating the following in your Competing Interests section: “NO”

Please complete your Competing Interests on the online submission form to state any Competing Interests. If you have no competing interests, please state "The authors have declared that no competing interests exist.", as detailed online in our guide for authors at http://journals.plos.org/plosone/s/submit-now.
This information should be included in your cover letter; we will change the online submission form on your behalf.

**Author response:** We declare that we have no competing interests.

4. We note that your Data Availability Statement is currently as follows:

“All relevant data are within the manuscript and its Supporting Information files.”

Please confirm at this time whether or not your submission contains all raw data required to replicate the results of your study. Authors must share the “minimal data set” for their submission. PLOS defines the minimal data set to consist of the data required to replicate all study findings reported in the article, as well as related metadata and methods (https://journals.plos.org/plosone/s/data-availability#loc-minimal-data-set-definition).

For example, authors should submit the following data:

- The values behind the means, standard deviations and other measures reported;
- The values used to build graphs;
- The points extracted from images for analysis.

Authors do not need to submit their entire data set if only a portion of the data was used in the reported study.

**Author response:** The sound recordings deposited on Xeno-canto were the source of data for this manuscript. These recordings are visually represented in the spectrograms. The images represent the entire length of the sound recordings, with the areas of higher intensity (darker red) used to assess the peak frequency and the duration of syllables. These were then used for the measurements of the mean, standard deviation, and standard error for the values reported. All data can be replicated with the deposited recordings. Additional recordings do exist and can be uploaded to Xeno-canto if requested; however, this manuscript is based on the uploaded recordings.

5. Please amend the manuscript submission data (via Edit Submission) to include author “Yelena Cerezke-Riemer”.

**Author response:** The author submission has been edited on for the manuscript submission.

6. Please ensure that you refer to Figure 5 in your text as, if accepted, production will need this reference to link the reader to the figure.

**Author response:** This reference has been added into the body of the manuscript.

7. Please review your reference list to ensure that it is complete and correct. If you have cited papers that have been retracted, please include the rationale for doing so in the manuscript text, or remove these references and replace them with relevant current references. Any changes to the reference list should be mentioned in the rebuttal letter that accompanies your revised manuscript. If you need to cite a retracted article, indicate the article’s retracted status in the References list and also include a citation and full reference for the retraction notice.

**Author response:** All referenced have been reviewed, with all additional referenced added.

**Response to Reviewer 1**

**Reviewer #1**: This manuscript describes the characteristics and occurrence of photosynthetic bubble stream sounds (POBS) from Sago pondweed as well as other sounds recorded in Gull Lake, Alberta, Canada. The authors provide visualizations and descriptions of five exemplar POBS recordings and discuss their attempts to identify or localize other sound sources in their study system. This type of observational or naturalist study provides valuable insights needed to untangle the diversity of sounds found in aquatic soundscapes as well as support future efforts to apply passive acoustic monitoring to what are often understudied underwater ecosystems. I found this study particularly interesting because it focuses on a less discussed category of biological sounds.

I appreciated the study and its efforts and I think it will be a valuable contribution to the scientific literature. The manuscript’s contents fall within the journal’s scope (i.e., by conducting original research contributing to our knowledge of underwater sound sources). As far as I could ascertain, the methods employed by the authors were valid and appropriate for their study objectives and their conclusions were generally reasonable and relevant to the work conducted. I especially appreciated their provision of multiple, diverse exemplar recordings with associated descriptions and measurements.

Though I appreciated the findings in the manuscript, I would have liked to see more detail provided in the methods, especially related to how the authors worked to identify the sources of sounds. For example, in the results on lines 63 and 79, the authors state they recorded corixids and a plant in aquaria to supplement their field recordings, but the aquaria testing is not described at all in the methods. They could also include a list of the sources they used for identifying previously documented sound producers anmore detail as to how the visual sound-producer assessments were conducted (e.g., were they in the water or on the shore or both, how many people were involved in the assessments). Similarly, I would appreciate if the authors could provide an idea of how many recordings at what lengths and over how many days and locations within the lakes were assessed as part of the study. Because the field of underwater bioacoustics has historically struggled with misidentified sound sources, I believe adding more information like this to the methods would aid readers’ confidence in the authors’ findings.

**Author response:** Thank you for these suggestions, this is a very helpful review. The methods section has been expanded and restructured in response to this request, and we hope that it clarifies exactly how the study was performed [lines 57-104].

Corixids with structures that appear to be capable of producing sound are listed by Hungerford [line 149]. Aquatic insects with structures that appear to be capable of producing sound are listed by Aiken [line 149]. Both of these references have been added.

For sampling effort, the number of recordings made in the shallows of Gull Lake in addition to the total number of recordings obtained, and the total minutes that these recordings represent have been added [lines 107-111].

I had several other recommendations related to the manuscript, outlined below, encompassing the possible inclusion of more acoustic measurement statistics and some writing corrections. I consider all of the revisions recommended relatively minor.

Lines 8, 170, and possibly elsewhere: Sentences shouldn’t begin with an acronym, so the acronyms should be either spelled out or the sentence should be reworded.

**Author response:** Agreed, all sentences that started with acronyms have been either changed or spelled out.

Lines 8, 76, 85, and possibly elsewhere: Sago is sometimes capitalized, sometimes not, so this should be made consistent throughout.

**Author response:** All instances of "sago pondweed" have been changed to lower case.

Line 46: There is a period missing at the end of this sentence.

**Author response:** Thank you, the period was added after the citation.

Line 88: The authors state in the table caption that they provide “average peak frequency” but then the table values themselves seem to be more reflective of ranges, so perhaps the caption should be corrected. Relatedly, however, if possible, I think it would be the most helpful to provide the mean, standard deviation or error, and ranges of the peak frequencies and durations from each of the exemplar recordings as different readers might prefer some or all of the values for different purposes. Additionally, there is not a widely used standard for referring to individual units of multi-part sounds, here referred to as “syllables.” Because of this, it might be worthwhile to define what is meant by syllable, either in the caption itself or in the methods.

**Author response:** Thank you for this comment. The table has been clarified to represent ranges of peak frequency. In addition, the standard deviation and standard error have been added for the duration and frequency ranges of the sounds [line 174]. A definition of syllable has been added to the methods section of the manuscript [lines 81-82]. This follows the terminology suggested by Baker and Chesterfield (2020) and this citation has been added [line 81].

Table 1, Line 111, and possibly elsewhere: When reporting a range of values, an en dash “–“ should be used rather than a hyphen.

**Author response:** All uses of hyphens for ranges have been changed to en dashes.

Line 98: I really appreciated the authors onomatopoetic descriptions here and elsewhere along with the more quantitative descriptions of the sounds.

**Author response:** Thank you for this, it is a good way to understand the description if the reader is not listening to the sound recordings while reading the paper.

Lines 170, 184, and possibly elsewhere: When referring to multiple fish species (rather than multiple fish individuals that are the same species), then the plural of “fish” should be “fishes”

**Author response:** This has been changed throughout the manuscript.

Line 171: I believe this reference citation should be changed from “Wilson 2004” to “30”.

**Author response:** This mistake in referencing format has been changed.

Line 191: I believe “soundscape” should be made plural to fit the grammatical sentence structure.

**Author response:** The plural of soundscape works much better here and has been changed.

Line 213 and elsewhere: The more commonly used name for these types of figures are spectrograms, whereas I believe spectrograph usually refers to something that splits light into its component colors. Also, from the figures, the authors could note here in the methods that they created both spectrograms and waveforms of their exemplar recordings.

**Author response:** All instances of spectrograph have been changed to spectrogram. The methods have been clarified as to the creation of the spectrograms and waveforms, which was done in Raven Lite2 [lines 78-81].

References: Though not a necessary inclusion, the authors may find this recent publication of interest: Desjonquères C, Linke S, Greenhalgh J, Rybak F, Sueur J (2024) The potential of acoustic monitoring of aquatic insects for freshwater assessment. Philos Trans R Soc B 379:20230109. Also, in glancing through the references, I did find a few errors, such as the use of title case instead of sentence case in reference 2, listing the journal of reference 31 as AFS instead of Fisheries, and an extra space after the year in line 27, so it may be worth checking them all again during revision.

**Author response:** Thank you for the reference suggestion, it has been added into the paper [line 148]. All references have been reviewed for errors in formatting.

**Response to Reviewer 2**

**Reviewer #2:** This is an interesting piece of research that quite rightly highlights the importance of sound production by submerged aquatic plants and provides a description of sound production by Stuckenia pectinata. However, I feel that there are some key pieces of information missing from the manuscript that should be added before publication. For example, I think that the introduction could be expanded slightly to include a few key papers (see suggestions below). Also, more detail is required in the methods section in general, and some methods appear in the results section (e.g., aquarium recordings and the use of gas chromatography) but are not described in the methods section. You mention that the recordings were uploaded to Xeno Canto in the results - I would also put this in a Data Availability Statement at the end of the manuscript. I also think that the discussion and conclusion could be improved by thinking about the wider implications for monitoring aquatic plant sounds. These could include the use of aquatic plant sounds as indicators of pollution events / measuring the primary productivity of an ecosystem. Also, many macrophytes are indicator species and are used to assess the ecological condition of an ecosystem. Perhaps the ability to identify key macrophyte species’ sounds will allow for the assessment of freshwater ecosystems using passive acoustic monitoring?

**Author response:** Thank you for these comments and time that was put into this review. It has helped immensely in the manuscript. A data availability statement has been included at the end of the manuscript [lines 303-306]. All of the suggestions for the introduction, details in the methods, and discussion expansion have been included.

**Abstract**

4: Most *of the* work on freshwater soundscapes.

**Author response:** This has been changed [line 3].

11: What are these important consequences for acoustic bioinventories exactly?

**Author response:** Ecosystem primary production measurements and rapid ecosystem assessments have been included here as possible important uses [lines 12-14].

**Introduction**

19 - Freshwater is one word (check for other instances throughout).

**Author response:** Freshwater has been changed to be one word throughout.

25 - Following “with some exceptions… add the following references that include descriptions of plant sounds in freshwater habitats:

Kratochvil, H. G., & Pollirer, M. (2017). Acoustic effects during photosynthesis of aquatic plants enable new research opportunities. Scientific Reports, 7(1), 44526.

Greenhalgh, J. A., Genner, M. J., & Jones, G. (2023). Diel variation in insect‐dominated temperate pond soundscapes and guidelines for survey design. Freshwater Biology, 68(7), 1148-1160.

**Author response:** Both of these citations have been included in the manuscript [line 27].

31: This is such a cool fact! I had no idea… Double check this reference though - It doesn’t seem to include anything on beetles. Perhaps this is better? <https://www.tandfonline.com/doi/abs/10.1080/01650424.2011.572558>

**Author response:** This reference has been changed to the one suggested here [line 34]. An additional reference as also been added (Larson et al., 2000) [line 34].

48: avoid ‘numerous’ and state the total number of hours/sites etc.

**Author response:** This has been corrected [lines 107-108].

48: The hypothesis / research aim should be more clearly defined than “To get a better sense of the soundscapes in shallow fresh water”. Is the aim of the manuscript just to describe pondweed sounds, or to describe the entire soundscapes of these lakes? It would be good to include some analysis of the water boatman sounds if you are taking the soundscape approach.

**Author response:** We have restated our hypothesis accordingly, although it was more like a research goal than a hypothesis sensu stricto [lines 51-55].

**Results**

65 - 67: This sentence should be moved to the discussion.

**Author response:** It has been moved to lines 274-277.

69: This should be moved to the discussion, also add a recent view here that suggests >7,000 aquatic insect species are likely to produce sound globally: Desjonquères, C., Linke, S., Greenhalgh, J., Rybak, F., & Sueur, J. (2024). The potential of acoustic monitoring of aquatic insects for freshwater assessment. Philosophical Transactions of the Royal Society B, 379(1904), 20230109.

**Author response:** The suggested reference has been included in this section [line 148]. The possibility of infrequent defensive stridulations has also been included [lines 276-277].

79: Can you explain how the aquarium recordings were made? How big was the tank? Where did you put the hydrophone? How did you ensure that no animals from the lake that might also produce sounds were still attached to the plant?

**Author response:** Yes, we have revised this section in response to the first reviewer as well [lines 91-97].

81: What were the other gases other than oxygen detected by the gas chromatography?

**Author response:** We were able to obtain the original lab results from Dr. Gingras, and they are included here now [lines 98-104]. Our original text was based on a verbal conversation, which we agree was inadequate.

150: Please consider uploading your sounds to The Freshwater Sounds Archive and be included as a co-author in an upcoming data paper: <https://fishsounds.net/freshwater.js>

**Author response:** All exemplar sounds have been uploaded to The Freshwater Sounds Archive.

**Methods**

Need to include methods regarding the laboratory recordings, gas chromatography, and making the acoustic measurements (peak frequencies etc).

**Author response:** The methods section [lines 57-104] has been expanded and restructured, to better explain exactly how the study was conducted.

207: Add sampling rate and gain used.

**Author response:** Sampling rate and gain settings have been added [lines 60-61].

207: How long did you record for? Can you be specific about the times of day that the recordings were made at? (Perhaps by checking the .wav file name time stamp?)

**Author response:** This section has been revised accordingly, adding the requested information [lines 85-87; 107-113].

213: Spectrograms, not Spectrographs (check for other instances throughout). Also add FFT size and % overlap used to generate the spectrograms.

**Author response:** This has been changed throughout, in addition to a short-time Fourier transform size with a 50% overlap parameter for the spectrogram creation [lines 62-63].

**Discussion**

155: Avoid “at our study site” and put something like: The shallow water soundscape of Lake Gull in Alberta was dominated by….

**Author response:** This has been changed throughout.

156: Personally, I am not a fan of making things into acronyms unless it’s absolutely necessary. I recommend removing them in favour of clear and simple language, but this is a stylistic thing and your choice.

**Author response:** Pending the editors' permission, we prefer to retain the acronym, since the full phrase is rather long, and the acronym would be at home in a field that also included FRTs and acronyms for various acoustic parameters.

159: Maybe add some clarity here and state that you think the bubbles are produced by incisions made in the leaf structure by aquatic insects, such as plant breathers (if this is indeed what you mean). Did you see this happening? It would be great to prove this. Or perhaps you mean by damage sustained by the plant? If this is the case then is it best to describe the bubbles as photosynthetic bubbles?

**Author response:** The manner in which the plant tissues were damaged was not observed; however, in the shallow water with only occasional wave action, numerous aquatic animals, and wading and swimming birds, it is reasonable to assume that damage to the plants could occur for a variety of reasons. This has been clarified on lines 32-24. Even if bubbles are escaping through wounds, they are still comprised of oxygen that is produced from photosynthesis, so we argue for retaining our preferred terms.

171: keep the referencing style the same.

**Author response:** This reference format has been changed.

173: The use of acronyms is confusing here. Make a clear distinction when you are talking about sounds produced by fish and sounds produced by plants.

**Author response:** FRT are produced only by fishes, and POBS are produced only by plants. It is not clear where the confusion arises here and we believe that the text is unequivocal.

187: See The Freshwater Sounds Archive: <https://fishsounds.net/freshwater.js>

**Author response**: See earlier response: we have uploaded files to this portal, and indicated this in the text.

190: Add Greenhalgh, J. A., Genner, M. J., & Jones, G. (2023). Diel variation in insect‐dominated temperate pond soundscapes and guidelines for survey design. Freshwater Biology, 68(7), 1148-1160.

**Author response:** This citation has been included in this section [line 279].

200: How about the potential to use aquatic plant sounds as indicators of pollution events / measuring the primary productivity of an ecosystem. Also, many macrophytes are indicator species and are used to assess the ecological condition of an ecosystem. Perhaps the ability to identify key macrophyte species’ sounds will allow for the assessment of freshwater ecosystems using passive acoustic monitoring?

**Author response:** The potential use for these sounds as indicators and measurements of primary production have been included [295-299]. We agree that there is also room for development in this technique in the future, allowing for increased use in species identification.

216: Can you be more specific about how these variables were calculated? Were they calculated in Raven 2? What tool did you use, to what decimal place etc…

**Author response:** More details were included here on how these were measured in Raven Lite2, and our visual assessment of the spectrograms [lines 80-82].

**Tables**

Include the species name in table legend. Add standard error values for each measurement.

**Author response:** The species name has been included in the caption. The standard error and standard deviation of the durations and peak frequencies (as available) have also been included [lines 169-174].

**Figures**

I think that the frequency range on the y axis should be standardised across all spectrograms to allow for direct comparison. Also, each figure legend needs to include the FFT size and overlap parameters used to create them. No need to explain the axis of each figure, the units do that for us.

**Author response:** The y-axis for the spectrogram figures have been standardised to 20 kHz, with the addition of the FFT size and overlap included in the caption.

Do you have any pictures of the pondweed and the lakes you sampled? It would be great to see what they look like.

**Author response:** This has been included as Photo 1 [lines 115-117].

**Response to Reviewer 3**

**Reviewer #3**: This study provides field characterization of an underappreciated component of freshwater soundscapes. I believe this should be published, as it will help progress efforts in freshwater bioacoustic monitoring. However, currently there is not enough detail in the methods and results to determine the initial goal of the study, how the soundscape was sampled, or back up the claim that bubble streams from macrophytes are a dominant part of the soundscape.

**Author response:** Thank you for the comments! They have been considered and were helpful in clarifying the methods used for the manuscript. The methods section has been expanded and restructured [lines 57-104].

**Introduction:**
Line 21: I see what you’re saying here, but the last two sentences of the first introduction paragraph initially seem to be contradictory. Simply swapping the order of sentences would make your point clearer and improve the flow into the next paragraph.

**Author response:** The order of these sentences has been swapped to make a stronger point and increase flow [lines 21-23].

Line 46: It is worth acknowledging that most sounds attenuate do not propagate well in shallow water, so this is not necessarily a characteristic of these specific signals. Soundscapes may be highly localized, but specifically for shallow-water organisms.
Relevant citations:
• Forrest, T. G., G. L. Miller, and J. R. Zagar. 1993. Sound propagation in shallow water: Implications for acoustic communication by aquatic animals. Bioacoustics 4:259–270.
• Fine, M. L., and M. L. Lenhardt. 1983. Shallow-water propagation of the toadfish mating call. Comparative Biochemistry and Physiology -- Part A: Physiology 76:225–231.

**Author response:** It has been clarified about attenuation of all sounds in shallow water and these citations have been included [lines 48-50].

**Methods:**
There should be much more detail here. It is not clear whether you were passively looking at the whole soundscape (stationary hydrophone with paired direct or camera-based observation through duration of recording) or actively characterizing possible components of the soundscape (selecting possible sound sources based on visual observation and placing a hydrophone near them).
There is almost no discussion of the analysis of the recordings, although it is discussed in the results. How were the visual assessments linked to the analysis of recordings? Were all recordings reviewed manually?

**Author response:** The methods section has been expanded and restructured, and we hope that it now satisfies this requirement [lines 57-104].

Line 205: How many cumulative minutes of recording did you obtain? What was the distribution of times of day? If you were actively seeking specific sound sources, please list which possible sound sources were targeted and how many recordings were taken of each.

**Author response:** The total number of recordings and the range of lengths were included**,** along with the cumulative number of minutes [lines 107-109].

Line 207: Was the hydrophone stationary or mobile? My impression is that you selected potential sound sources and placed the hydrophone to record potential sounds (in the results, you mention following a school of fish), but that is not clear here. Please also note approximate position of the hydrophone in the water column- obviously most of your recording regions were very shallow, but it would be nice to know whether the hydrophone depth was at surface, bottom, or varied to match the sound sources. If you recorded specific sound sources, please note how many recordings were taken for each potential sound source, and possibly the variation in time of day of recording (especially for macrophytes!). Please also note the sample rate of the hydrophone.

**Author response:** The hydrophone was mobile, held by the cable by hand. We have explained this in the text [lines 64-76].

Line 209: More detail needed on visual assessments as well. Were both cameras and direct observation used for each recording? Were all possible sound sources noted for each recording? How exactly were the visual assessments and recordings linked?

**Author response:** We have clarified this as well [lines 68-77].

Line 213: All uses of “spectrograph” should be replaced with “spectrogram”

**Author response:** All used of spectrographs have been changed.

Line 216: How were the peak frequencies and duration of repeated sections characterized?

**Author response:** The peak frequencies and durations of sections were manually determined in Raven Lite2. This has been clarified in the methods [lines 76-81].

Line 218: Unclear what “additional recordings were also considered” means, especially since I do not see them in the results. If they were analyzed for soundscape components, please describe the methods of recording and analysis. If they were not analyzed in a way that could be used in the results, they should not be in the methods.

**Author response:** This has been clarified [lines 88-90].

**Results:**
As noted, there was no discussion of the analysis in the methods that yielded the first paragraph of the results. As such, it is difficult to interpret statements like the first sentence- were they simply the only sound source in most of the recordings, or were they stronger in volume? Were these sounds found in most recordings? It would be informative to have some more numbers here. If the hydrophones were placed specific to a given potential sound source, what percentage of the recordings actually yielded a distinctive signal? If the hydrophones were placed randomly to capture an entire soundscape, what percentage of recordings featured each type of sound, and how did the occurrences of a visually observed sound source align with the occurrences of its associated sound? When multiple sound sources were present, which was higher in volume?
I would recommend some restructuring of this section to first enumerate what species were present, then how often each produced sounds.

**Author response:** The proportion of signals across all recordings considered that were plant sounds and corixids has been included in this section [lines 107-109].

Line 57: Are you certain these are methane bubbles?

**Author response:** The production of methane bubble production is typical of lakebed sediments; however, these bubbles were not analysed. For our purposes, the mode of formation of the craters (also known as pocket marks, or pock marks) is not important.

Line 60: Were boats present during recordings? As above, some description of the comparisons between your visual and auditory surveys would contribute greatly to this section.

**Author response:** Boats were often visible, but inaudible through the hydrophone. We assume that the sandy substrate and sand ripples dampen the boat sounds, and results in none of them reaching the shallow zone in which we worked. The visual and auditory comparison has been discussed above [lines 159-163].

Line 63: Aquaria recordings were not described in the methods.

**Author response:** There has been a clarification, and more details added pertaining to the aquarium recordings [lines 91-97].

Line 64: Were abundances of insects anecdotally observed or quantified in any way? If there were more potential sound makers than were notably absent from the recordings, please include all of their names.

**Author response:** We have added some discussion of this issue [lines 143-147; 150-163].

Line 73: If there were more fish species observed that did not make sounds, please list them; if not, change wording of sentence to reflect that these are all fish that were observed in this study.

**Author response:** These were the only species of fish seen in the shallows, and this has been clarified [lines 156-158].

Line 79: How was the aquarium plant “roughly comparable” to Exemplar 3? Similar in frequency distribution, similar in pulse rate?

**Author response:** This sound was comparable to exemplar 3 as it had the same sound pattern produced at a similar frequency. This has been clarified [lines 129-130].

Line 85: Inconsistent capitalization of Sago/sago.

**Author response:** We have changed to lower case throughout.

Line 95: Since the analysis was not described in the methods, I can’t tell how all of these descriptive measures were decided. Did you measure each syllable individually? If so, then “duration of syllables” in the table should be mean duration of syllables, and it would be nice to include a measure of spread (standard deviation, etc.). This also seems like the minimum and maximum peak frequencies from the spread across each syllable, but it’s hard to determine.

**Author response:** More clarification has been added in the methods, including the manual assessment of these characteristics. The mean duration of syllables has been included, along with the range of peak frequency. Standard deviation and standard error have also been included were possible [lines 174].

Line 122: Unless part of the signal that was analyzed is not in the figure, I don’t see what part of this signal would likely have had a peak frequency of 6.2 kHz.

**Author response:** This is the spread of the peak frequency, which is being interpreted as the red sections of the spectrogram. In Exemplar 3, this ranges from 0.9 kHz to 6.2 kHz.

Line 147: Should be Exemplar 4

**Author response:** Noted and changed.
**Discussion:**
Line 155: I do not see enough evidence in the results to support this claim. Please back this up with how the prevalence of the bubble streams compared to other signals. There was also no further discussion of the corixid stridulations beyond the first sentence of the results section. Even if the corixid stridulation has been described in other papers, it would be beneficial to include a description of the characteristics of the stridulation signal in this setting.

**Author response:** The prevalence of the bubble streams have been clarified [lines 241-243]. There has been more discussion of the corixid sounds [lines 268-277].

Line 158: Fully submerged plants typically do not have stomata, and I believe this is the case for sago pondweed. This does not negate the probability of these bubble streams being from wounds, but any “pearling” would not be associated with stomata on these plants.

**Author response:** Reference to the stomata has been removed.

Line 164: Acoustic ecologists are increasingly using machine learning algorithms to increase the efficiency of reviewing acoustic recordings. Can you describe any commonalities of the bubble streams that may be helpful for programming automated detectors? Additionally, you noted that Exemplar 4 was fairly distinct among the sounds. Were there characteristics of the plant or bubble stream that might explain this?

**Author response:** This is a good question; but we feel that the answer lies beyond our understanding. For the characteristics that make exemplar 4 sound distinct, we can only assume that the nature of the plant tissue, the wound, and the availability of oxygen would influence the sound. Hopefully, as more researchers become aware of these sounds, the answer to this question will come into focus.

Line 169: There are many other sound sources and types noted across all aquatic habitats- marine invertebrates, tonal fish calls, etc. This sentence is likely more accurate with respect to freshwater habitats.

**Author response:** This sentence has been changed to only apply to freshwater habitats.

Line 171: Citation formatting

**Author response:** The formatting has been changed.

Line 188: Good points here

**Author response:** Thank you!

Line 192: I think it would be worth acknowledging that all the bubble stream signals in this paper are from the same type of plant. Is this plant sufficiently ubiquitous in lakes in Canada that this is widely relevant? Are these bubble streams likely to be similar among freshwater species? What is the depth limit of Sago pondweed?

**Author response:** This has been added, with clarification on the source of the signals, the geographic range of sago pondweed, and the habitats in which it is found [lines 287-291]. Similar sounds were associated with other macrophytes, but this was not investigated any further by us.

**Figures:**
The font is small and a bit grainy- is there a way to get higher resolution, at least on the text? An image of a bubble stream might also make a good addition to your figures, especially since you make a distinction between a bubble stream from a wound and "pearling".

**Author response:** The text has been changed on the figures to be higher resolution. An image of the bubble streams has also been added in as Photo 2 [lines 135-136].
